# Sex-related differences in long-term tolerability of Risperidone ISM treatment in adult patients with schizophrenia

**DOI:** 10.1192/j.eurpsy.2025.716

**Published:** 2025-08-26

**Authors:** J. Martínez González, C. Sherifi, L. Anta Carabias, M. Almendros Gimenez, C. Salazar García, C. U. Correll

**Affiliations:** 1 Medical Department, Laboratorios Farmaceuticos ROVI S.A., Madrid, Spain; 2 Medical Affairs, ROVI Biotech Ltd., Croydon, United Kingdom; 3 Department of Psychiatry and Molecular Medicine, Donald and Barbara Zucker School of Medicine at Hofstra/Northwell, Hempstead, NY; 4 Department of Psychiatry Research, The Zucker Hillside Hospital, Glen Oaks, NY, United States; 5 Department of Child and Adolescent Psychiatry, Charité Universitätsmedizin Berlin, Berlin, Germany; 6 Center for Psychiatric Neuroscience, The Feinstein Institute for Medical Research, New Hyde Park, NY, United States; 7 German Center for Mental Health, Partner Site Berlin, DZPG, Berlin, Germany

## Abstract

**Introduction:**

Sex-related differences in antipsychotic treatment exist with some specific differences having been reported with risperidone use. Women may respond better to antipsychotics than men, but also experience more side effects. In a randomised controlled trial of 1460 participants, a significantly higher proportion of female than male study participants treated with risperidone reported greater rates of gynecomastia/galactorrhoea and incontinence/nocturia. There is a call to consider sex differences in mental health research [Galbally et al. CNS Drugs 2024; 38(7):559-570]. Risperidone ISM (Risp-ISM) is a monthly long-acting injectable (LAI) formulation of risperidone, recently authorised in Europe, USA and some other countries.

**Objectives:**

To investigate the sex-related differences in the safety of Risp-ISM in patients with schizophrenia during the PRISMA-3 study.

**Methods:**

A post-hoc analysis was performed on the PRISMA-3, 12-month open-label extension (OLE) study (NCT03870880) data [Filts et al. Schizophr Res. 2022; 239:83-91 [published correction in Schizophr Res. 2022; 246:258-259]].

**Results:**

215 patients received at least one dose of Risp-ISM. 84 (39%) were female. 66 (78.57%) females and 100 (76.33%) males experienced a treatment-emergent adverse event (TEAE). A lesser proportion of treatment-related (TR) TEAEs were experienced by females (F:45%; M:55%).

Overall, the most frequently reported (in ≥2% cases) TR-TEAEs are shown in Figure 1 and those leading discontinuation in Table 1. No cases of incontinence/nocturia were reported in either sex.

**Image 1:**

**Figure:**
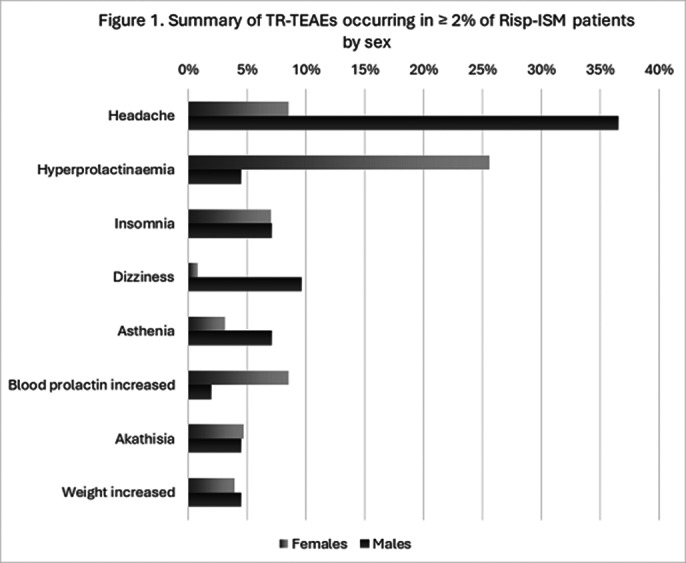

**Image 2:**

**Figure:**
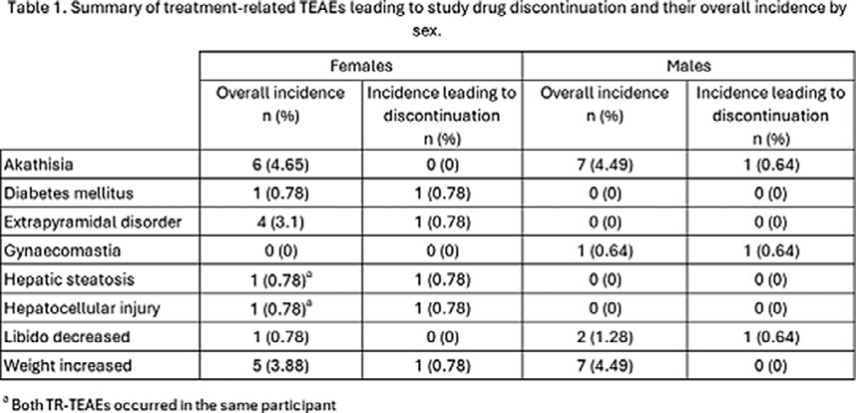

**Conclusions:**

Relating to the use of Risp-ISM, a lesser proportion of TR-TEAEs were experienced by females than males (45% vs 55%). Male participants were more likely to report dizziness and headache. Females were more likely to report raised blood prolactin and hyperprolactinaemia. This is in line with the findings in a large study which reported a significantly greater increase in prolactin levels among female participants [Galbally et al. CNS Drugs 2024; 38(7):559-570]. Very few participants discontinued Risp-ISM following a TR-TEAE (7/215 [3.3%]) during this 12-month extension phase [Filts et al. Schizophr Res. 2022; 239:83-91. [published correction in Schizophr Res. 2022; 246:258-259]].

**Disclosure of Interest:**

J. Martínez González Employee of: Laboratorios Farmaceuticos ROVI S.A., C. Sherifi Employee of: ROVI Biotech Ltd., L. Anta Carabias Employee of: Laboratorios Farmaceuticos ROVI S.A., M. Almendros Gimenez Employee of: Laboratorios Farmaceuticos ROVI S.A., C. Salazar García Employee of: Laboratorios Farmaceuticos ROVI S.A., C. Correll Shareolder of: Cardio Diagnostics, Kuleon Biosciences, LB Pharma, Mindpax, and Quantic., Grant / Research support from: Janssen and Takeda., Consultant of: AbbVie, Acadia, Alkermes, Allergan, Angelini, Aristo, Biogen, Boehringer-Ingelheim, Cardio Diagnostics, Cerevel, CNX Therapeutics, Compass Pathways, Darnitsa, Denovo, Gedeon Richter, Hikma, Holmusk, IntraCellular Therapies, Jamjoom Pharma, Janssen/J&J, Karuna, LB Pharma, Lundbeck, MedAvante-ProPhase, MedInCell, Merck, Mindpax, Mitsubishi Tanabe Pharma, Mylan, Neurocrine, Neurelis, Newron, Noven, Novo Nordisk, Otsuka, Pharmabrain, PPD Biotech, Recordati, Relmada, Reviva, Rovi, Sage, Seqirus, SK Life Science, Sumitomo Pharma America, Sunovion, Sun Pharma, Supernus, Takeda, Teva, Tolmar, Vertex, and Viatris.

